# Assessment of Cervicocephalic–Peripheral Atherosclerotic Burden Improves Prognostic Stratification in Patients with Ischemic Cerebrovascular Disease

**DOI:** 10.3390/jcm14186593

**Published:** 2025-09-18

**Authors:** Lu-Guang Li, Xin Ma, Xiaoxi Zhao, Xiangying Du, Chen Ling

**Affiliations:** 1 Department of Neurology, Xuanwu Hospital, Capital Medical University, Beijing 100053, China; luguang1997@outlook.com (L.-G.L.); zhaoxxmy@163.com (X.Z.); 2 National Clinical Research Center for Geriatric Disorders, Beijing 100053, China; duxying_xw@163.com; 3 The Neuro Cardio Vascular Diseases Center, Xuanwu Hospital, Capital Medical University, Beijing 100053, China; 4 Department of Radiology, Xuanwu Hospital, Capital Medical University, Beijing 100053, China; 5 Department of Vascular Ultrasound, Xuanwu Hospital, Capital Medical University, Beijing 100053, China; lingchenwu33@sina.com

**Keywords:** ischemic cerebrovascular disease, atherosclerosis, renal artery stenosis, prognosis, peripheral arterial disease

## Abstract

**Background:** Concurrent atherosclerosis (AS) in peripheral arteries may worsen the prognosis of ischemic cerebrovascular disease (ICVD) patients. Although cervicocephalic atherosclerotic burden (AB) has demonstrated strong risk stratification capabilities, whether peripheral arterial evaluation provides incremental prognostic value remains unclear. This study aimed to determine whether cervicocephalic–peripheral AB (CPAB) improves risk stratification in ICVD patients. **Methods:** This prospective cohort study consecutively included acute ICVD patients. AB scores for intracranial, cervical, renal, and lower extremity arteries were assigned as 0 (no stenosis), 1 (significant stenosis in one segment), or 2 (significant stenosis in ≥2 segments). The total score (range 0–8) was trisected into low, medium, and high CPAB levels. The primary endpoint was a composite of ischemic stroke, acute coronary syndrome, and vascular death. Model performance was evaluated using Harrell’s C and Somers’ D. **Results:** Among 403 patients (mean follow-up: 9.6 ± 5.4 months), 41 primary endpoints occurred, and 21 (5.2%) were lost to follow-up. Of 382 patients analyzed, 30.6% had significant peripheral AS. Patients with concurrent peripheral-cervicocephalic AS had a higher risk of vascular events (*p* = 0.001) than those with single-territory AS. CPAB was independently associated with the primary endpoint (HR = 2.22, *p* < 0.001) and stroke recurrence (HR = 1.90, *p* = 0.013). While cervicocephalic AB also independently predicted outcomes, the CPAB-based multivariate Cox model had improved discriminative performance (Harrell’s C = 0.678 vs. 0.653 for primary endpoint, *p* = 0.02; 0.646 vs. 0.634 for stroke recurrence, *p* = 0.03). **Conclusions:** Peripheral AS is common in ICVD patients and contributes independently to vascular risk. The CPAB score, which integrates atherosclerotic burden from both cervicocephalic and peripheral territories, could improve prognostic stratification compared to single-territory or cervicocephalic AB alone, supporting comprehensive multiterritorial AS assessment to guide risk-based management strategies.

## 1. Introduction

As a systemic disease, atherosclerosis (AS) not only involves cervicocephalic arteries, leading to ischemic cerebrovascular disease (ICVD) that poses a major threat to human health [1], but also frequently affects various other vascular beds throughout the body [2]. Peripheral arteries are among the most commonly affected sites of AS. Approximately 10% of elderly individuals suffer from peripheral atherosclerotic diseases (PAD), primarily lower extremity arterial disease (LEAD) and renal artery stenosis (RAS) [3]. The prevalence of these diseases in ICVD patients is about 25%, significantly higher than in the general population [4]. PAD is associated with a significantly elevated risk of cardiovascular events and mortality [5], and patients with multiterritorial AS also generally have an increased incidence and mortality rate of ischemic stroke (IS), myocardial infarction (MI), and other vascular events [6]. Recent guidelines suggest that patients with multiterritorial AS may require more stringent risk factor control strategies [3]. Moreover, differences exist between antiplatelet therapy regimens for ICVD patients and PAD patients [3,7]. However, PAD screening in ICVD patients has not been widely implemented. Additionally, research on the impact of LEAD and RAS on the prognosis of ICVD patients remains relatively scarce, and the association between their severity and IS recurrence has not been fully established [8,9].

Atherosclerotic burden (AB) provides a method to simultaneously assess both the severity and extent of AS. Our previous studies demonstrated that cervicocephalic AB offered improved risk stratification for vascular events in ICVD patients compared to evaluating AS in isolated arterial segments [10,11], and a comprehensive assessment of multiterritorial AB could also provide more prognostic information than evaluating single-territory AB [12]. A recent study preliminarily proved that patients with a higher total AB across major vascular territories have a greater incidence of vascular events. However, the proposed AB assessment method is difficult to implement on a large scale since it relied on digital subtraction angiography (DSA), and it did not address whether the assessment of peripheral arterial territories contributes additional prognostic value beyond cervicocephalic AB [13]. Computed tomography angiography (CTA) is widely used in the examination of cervicocephalic AS, while arterial ultrasound, characterized by high specificity and sensitivity, is a non-invasive and convenient first-line method recommended by guidelines for screening AS in lower extremity and renal arteries [3]. Using CTA and ultrasound to evaluate AS status in ICVD patients, we quantitatively assessed the single-territory AB of cervical, intracranial, renal, and lower extremity arteries, and the overall cervicocephalic–peripheral AB (CPAB) based on these four single-territory Abs. This study aims to explore the relationship between ABs in different arterial territories and the risk of stroke recurrence and vascular events, and to establish and validate a simple, non-invasive CPAB assessment method to facilitate improved risk stratification and inform clinical decision-making for ICVD management.

## 2. Materials and Methods

This study is a prospective cohort investigation. All participants provided informed consent before enrollment. Ethical approval for all research procedures was granted by the Ethical Committee of Xuanwu Hospital and conducted in accordance with the Helsinki Declaration. Data supporting the findings of this study can be obtained from the corresponding author upon reasonable request by researchers.

### 2.1. Study Population and Data Collection

We consecutively enrolled ICVD patients admitted to Xuanwu Hospital from June 2020 to July 2022. IS cases were categorized based on the TOAST criteria for stroke etiology [14]. Inclusion criteria were age between 18 and 80, AS-related IS (large-artery AS or small-artery occlusion subtype according to the TOAST classification) or transient ischemic attack onset within 30 days confirmed by head CT or MRI. Exclusions included non-atherosclerotic arterial stenosis (arterial dissection, arteritis, etc.), cardioembolism stroke, stroke of undetermined etiology according to the TOAST classification [14], those who had a history of cervicocephalic revascularization, and individuals unable to complete required examinations.

Baseline data and medical history included age, gender, body mass index (BMI), history of hypertension, diabetes, hyperlipidemia, smoking, alcohol use, ICVD history, and coronary heart disease history. Blood tests on the second day of admission covered glucose, glycated hemoglobin, total cholesterol, triglycerides, low-density lipoprotein, high-density lipoprotein, homocysteine, C-reactive protein, apolipoprotein A1, apolipoprotein B, fibrinogen, D-dimer, neutrophil count, creatine kinase, and creatinine. Neurological deficit severity of patients was evaluated based on the National Institute of Health Stroke Scale.

### 2.2. Assessment of Atherosclerotic Characteristics

All patients underwent cervicocephalic CTA and peripheral artery ultrasound examinations to screen for AS within 7 days of admission. The cervicocephalic arteries were classified as cervical (bilateral subclavian, common and extracranial carotid, extracranial vertebral; 8 segments in total) and intracranial (bilateral intracranial carotid, intracranial vertebral, anterior, middle, posterior cerebral, and basilar; 11 segments in total) arteries [15]. Cervicocephalic CTA scans were conducted using dual-source 192-layer spiral CT (LightSpeed, Montreal, QC, Canada, General Electric Company, Boston, MA, USA), and image reviews were performed by two radiologists unaware of clinical data. Bilateral lower limb arteries, including the common, superficial, and deep femoral, popliteal, anterior tibial, posterior tibial, and peroneal arteries (14 segments in total), and bilateral renal arteries (2 segments in total) were assessed by two ultrasound technicians using Philips IU22 and HDI5000 (Philips Medical Systems, Bothell, WA, USA) in a double-blind manner.

Significant AS in cervicocephalic or lower limb arteries was defined as the most severe stenosis within any segment of the region being ≥50% or occlusion, and it was defined as a peak systolic velocity ≥180 cm/s in the affected segment of the artery or occlusion in renal arteries [16]. Patients were diagnosed with CAS, LEAD, or RAS when significant AS was detected in the respective regions.

To simultaneously assess the severity and extent of AS, the single-territory AB for intracranial, cervical, renal, and lower extremity arteries was assessed using the following scoring criteria for arterial lesions: 0 for no significant atherosclerotic lesion, 1 for a significant atherosclerotic lesion in a single arterial segment, and 2 for significant atherosclerotic lesions in multiple arterial segments. The comprehensive score for CPAB followed the multiterritorial AB scoring method from our previous study [12], which involved summing up the four single-territory AB scores, resulting in a score ranging from 0 to 8; then, the total score was further trisectioned to a triple rank CPAB: low (0–1 points), mean (2–3 points), and high (>4 points). Using the same approach, the sum of cervical and intracranial AB was also trisectioned to a triple rank as the cervicocephalic AB (low: 0 points, mean: 1–2 points, high: 3–4 points) to evaluate the predictive efficacy of CPAB and cervicocephalic AB for vascular events of ICVD patients.

### 2.3. Follow-Up and Prognosis

Every 6 months, a physician unaware of the baseline data conducted telephone follow-ups with all enrolled patients to gather information on relevant medication use and key outcome events. Identified events were further confirmed by cardiologists or neurologists. The primary endpoint included recurrent IS, acute coronary syndrome, and death caused by cardio or cerebral vascular events. Patients out of contact during the follow-up were censored at the last time point when they were successfully connected, and the final follow-up was completed in December 2022.

### 2.4. Statistical Methods

Statistical analyses were performed using SPSS (v19.0; IBM, Armonk, NY, USA), Stata/MP (v17.0; www.stata.com), and R (version 4.3.0, R Foundation). All tests were two-tailed, and *p* < 0.05 was considered statistically significant. Normally distributed continuous variables were presented as mean ± standard deviation (X ± S), categorical variables as counts (%), and non-normally distributed continuous and ordinal variables as median and interquartile range [M (Q25, Q75)]. Missing values in the original data were imputed using the predictive mean matching method from the “mice” package in R. Univariate analysis was conducted using the χ^2^ test. The cumulative survival rates of endpoint events were estimated using the Kaplan–Meier method, and between-group comparisons were made using the Log-Rank test. Variables with statistical significance (*p* < 0.1) in the univariate Cox regression model were included in the multivariate Cox regression model to obtain the relative risk (RR) and 95% confidence interval (CI) to assess independent risk factors for the prognosis of ICVD patients. The predictive value of CPAB was further examined by adjusting for four single-territory ABs. Multivariable forward stepwise Cox regression analyses were separately performed including the four single-territory ABs, CPAB, or cervicocephalic AB, as well as related risk factors, and two different consistency indices (Harrell’s C and Somers’ D) were calculated to compare the predictive accuracy of different Cox regression models based on different AB scoring methods.

## 3. Results

### 3.1. Baseline Data and Outcomes

A total of 498 ICVD patients met the inclusion criteria, and 54 patients were excluded based on exclusion criteria; 41 patients either refused or were unable to complete relevant examinations. Ultimately, clinical data from 403 cases were complete and included by researchers. There were 21 patients (5.2%) lost to follow-up and 382 (94.8%) patients completing follow-up that were included in the prognosis analysis; baseline characteristics of patients lost to follow-up did not differ significantly from those retained. The average follow-up time was 9.6 ± 5.4 months. The mean age was 63 (55–69) years, and the patients were predominantly men (69.6%). There were 334 (87.4%) patients with IS, 195 with large-artery atherosclerosis stroke and 139 with small-artery occlusion stroke, and 48 (12.6%) patients with transient ischemic attack. All patients received standard antiplatelet therapy, statin therapy, and treatment to control risk factors, and at the end of the follow-up, the overall incidence rate of the primary endpoint was 10.7% (*n* = 41), including 26 (7.1%) non-fatal recurrent IS, 10 (2.6%) acute coronary syndrome, and 5 (1.3%) fatal cerebrovascular events (1 IS, 4 intracerebral hemorrhages). Older age, lower levels of high-density lipoprotein, and elevated creatine kinase levels were associated with an increased risk of vascular events (Table 1).

The occurrence of vascular events varied among patients with AS in different vascular beds. Patients with concurrent RAS had the highest incidence of primary endpoints and IS recurrence, followed by those with CAS and LEAD. Patients without significant AS had the lowest incidence of vascular events (Table 2). The incidence of vascular events was higher in multiterritorial AS patients (≥2) than patients with single-territory or without AS (Log-Rank *p* = 0.001). Patients with concurrent involvement of peripheral (renal or lower extremity) and cervicocephalic (intracranial or cervical) vascular beds also had a higher incidence of vascular events compared to those with single peripheral or cervicocephalic AS (Log-Rank *p* = 0.001).

### 3.2. Single-Territory AB and Its Relationship with Vascular Risk

**Figure 1:**
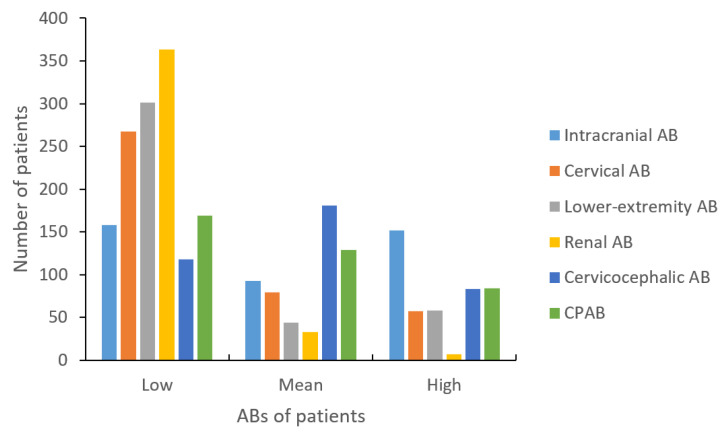
Single- and multiple-territory AB evaluated with different methods. Abbreviations: AB = atherosclerotic burden; CPAB = cervicocephalic–peripheral atherosclerotic burden.

Among the 382 patients, 129 (33.8%) had significant carotid AS, and 233 (61.0%) had significant intracranial AS. Additionally, 30.6% of patients had significant peripheral AS, including 98 (25.7%) patients with significant LEAD and 37 (9.7%) patients with significant RAS. The distribution of single-territory ABs for intracranial, cervical, lower limb, and renal arteries were different (Figure 1). Patients tended to have higher intracranial AB scores (average: 1.0), while average cervical (0.5), lower extremity (0.4), and renal (0.1) AB scores were lower. The relationship between different single-territory AB and the risk of vascular events and recurrent IS also varied. An increase in lower extremity (Log-Rank *p* = 0.010), renal (Log-Rank *p* = 0.006), intracranial (Log-Rank *p* = 0.002), and cervical (Log-Rank *p* = 0.032) AB was associated with an increased risk of vascular events. However, while there was a trend of increasing IS recurrence rates among patients with higher levels of the four single-territory ABs, only cervical AB was significantly associated with recurrent IS (Log-Rank *p* = 0.027).

### 3.3. ABs for Vascular Risk Stratification

**Figure 2:**
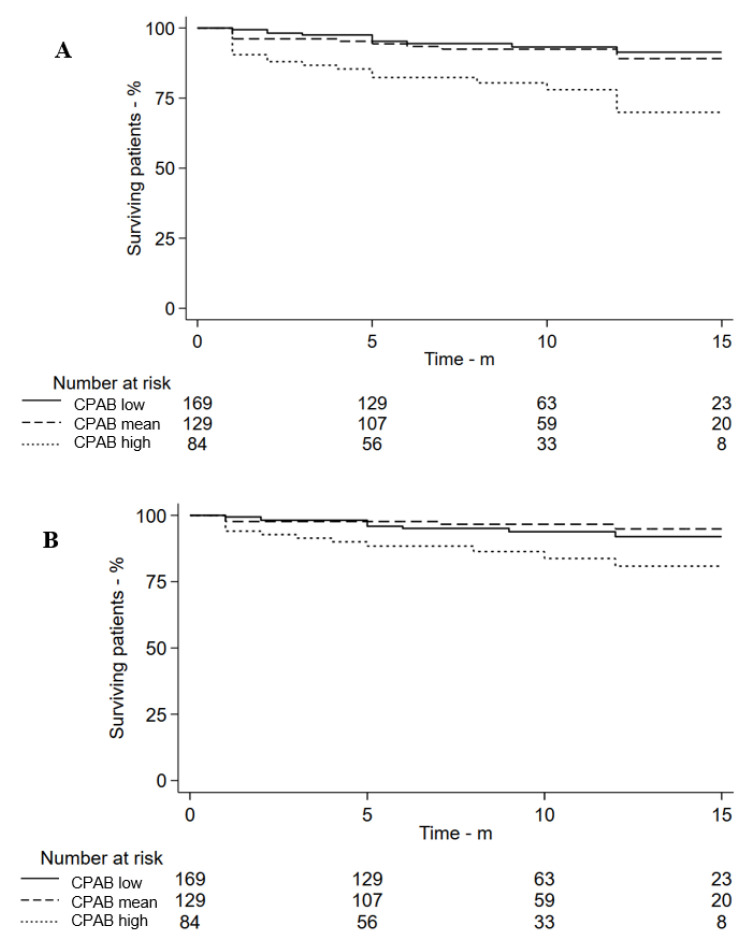
Cumulative survival curves for main outcome (**A**) and stroke recurrence (**B**) of CPAB in patients. Abbreviations: CPAB = cervicocephalic–peripheral atherosclerotic burden.

An increase in CPAB was associated with an increased risk of vascular events (Log-Rank *p* < 0.001) (Figure 2A) and IS recurrence (Log-Rank *p* = 0.008) (Figure 2B). When separately including the single-territory ABs, cervicocephalic AB or CPAB, as well as indicators associated with an increased risk of vascular events in univariate Cox regression (Table 1) into the multivariable Cox regression analysis, results showed that except for lower extremity AB which was not independently associated with stroke recurrence, each single-territory AB, cervicocephalic AB, and CPAB was independently associated with the primary endpoint and stroke recurrence (Table 3). However, when including four single-territory ABs, CPAB, and related risk factors simultaneously into multivariate COX regression, results showed that only CPAB was an independent risk factor for the primary endpoint (*p* < 0.001, HR = 2.22, 95% CI: 1.46–3.37) and recurrent IS (*p* = 0.013, HR = 1.90, 95% CI: 1.14–3.17), and four single-territory ABs could not independently predict the primary endpoint and stroke recurrence. Incorporating atherosclerotic characteristics into the multivariate Cox regression analysis, the results showed that CPAB’s predictive ability for vascular events is independent of intracranial artery stenosis (*p* = 0.004, HR = 2.07, 95% CI: 1.26–3.40), carotid artery stenosis (*p* = 0.023, HR = 1.79, 95% CI: 1.08–2.99), LEAD (*p* = 0.014, HR = 1.98, 95% CI: 1.15–3.43), and RAS (*p* = 0.004, HR = 2.02, 95% CI: 1.29–3.17).

Using the same method including single-territory ABs, cervicocephalic AB, and related risk factors (Table 1) into multivariate COX regression, results showed that cervicocephalic AB was also an independent risk factor for the primary endpoint (*p* = 0.002, HR = 2.05, 95% CI: 1.29–3.24) and recurrent IS (*p* = 0.02, HR = 1.93, 95% CI: 1.09–3.41) (Table 3). But as for the predictive efficacy of different ABs on vascular events, two consistency indicators, Harrell’s C and Somers’s D, both indicated that the multivariable Cox regression model based on CPAB (primary endpoint Harrell’s C = 0.678, Somers’s D = 0.357, IS recurrence Harrell’s C = 0.646, Somers’s D = 0.292) and related risk factors had a stronger risk assessment ability for the vascular events not only compared to the COX model based on single-territory ABs but also compared to the COX model based on cervicocephalic AB and related risk factors (primary endpoint Harrell’s C = 0.653, Somers’s D = 0.307, *p* = 0.02, IS recurrence Harrell’s C = 0.634, Somers’s D = 0.268, *p* = 0.03) (Table 3).

### 3.4. Sensitivity Analysis

Since four patients that died from intracerebral hemorrhage were included in the primary endpoint, and intracerebral hemorrhage differs pathologically from ischemic stroke, we conducted a sensitivity analysis of the primary endpoint after excluding these four patients (*n* = 378). In the multivariable Cox regression analysis that included CPAB, age, sex, and variables associated with increased vascular event risk in the univariable analysis, CPAB remained an independent risk factor for the primary endpoint (*p* < 0.001, HR = 1.36, 95% CI: 1.14–1.64). Moreover, the model incorporating CPAB demonstrated better predictive performance than models based solely on single-territory AB or on CAB (Harrell’s C = 0.674 vs. 0.622; Somers’s D = 0.348 vs. 0.253).

## 4. Discussion

AS frequently involves multiple vascular beds, and the incidence of LEAD and RAS in ICVD patients is much higher than in the general population [2,17]. However, the impact of certain peripheral arterial segments, particularly RAS, on the prognosis of ICVD patients remains controversial [8,9]. In this study, we used a reported method that uses significant stenosis to calculate the overall AB across different vascular beds [13] because mild stenosis may not significantly impact the hemodynamics of the affected area [18]. Moreover, we did not limit our evaluation to plaque burden or vulnerable plaque in individual arterial segments. While significant stenosis and plaque burden are collinear and both can independently predict vascular events, incorporating the distribution and location of lesions can further improve the assessment of vascular risk [19], whereas merely evaluating unstable plaques does not significantly enhance risk prediction [20]. Although a recent study reported that patients with a higher total AB across major vascular beds—including the cervicocephalic and peripheral arteries—had a greater incidence of vascular events [13], the invasive DSA method applied to evaluate AB is difficult to be widely used in clinical practice, and the sample size is small (*n* = 153). Further studies with large samples and non-invasive angiography to evaluate AB are needed. More importantly, the study did not compare total AB with single vascular bed AB to further understand its value in predicting vascular risk, and the role of peripheral AS in total AB is unclear. Lower extremity arterial ultrasound is one of the guideline-recommended first-line methods for LEAD screening, with both sensitivity and specificity exceeding 90% [21]. Similarly, renal artery ultrasound is also recommended by guidelines as the preferred method for RAS screening, with a PSV threshold of 180 cm/s suggested for the diagnosis of moderate stenosis [22]. In addition, compared with CTA, ultrasound is more cost-effective in the assessment of PAD [23]. Therefore, our study quantitatively assessed the severity and extent of AS in the cervicocephalic and peripheral arteries through non-invasive cervicocephalic CTA as well as peripheral artery ultrasound, calculated the CPAB, and compared the predictive effects of different AB measures on vascular events. Our study showed that while single-region AB and cervicocephalic AB alone could predict vascular events, the inclusion of peripheral arteries provided incremental prognostic value. These findings indicate that LEAD and RAS could contribute to the adverse outcomes of ICVD patients, supporting refinements to the existing multiterritorial AB model and underscoring the potential importance of comprehensively assessing multiregional AS to enhance risk stratification and improve prognosis.

As the most prevalent peripheral arterial atherosclerotic disease in ICVD patients [24], LEAD correlates with an increased risk of stroke recurrence, cardiovascular events, or vascular death [25]. Several studies have reported that ICVD patients with LEAD had a worse prognosis [26,27]. Our study reached the same conclusion and further confirmed that ICVD patients with more severe LEAD had worse prognoses. Ischemia related to LEAD contributes to an increase in mitochondrial DNA mutations and decreased activity, resulting in a mass and volume decrease in leg muscle [28]. It can also cause skin microcirculation disorders leading to ulcers, gangrene, and an increased risk of amputation. The resulting decline in lower limb function is associated with a heightened incidence of cardiovascular events and mortality [28]. Some research has also suggested that patients with combined LEAD have more severe traditional AS risk factors (such as higher blood pressure and glucose levels), elevated inflammatory markers (interleukin-6, tumor necrosis factor-α, fibrinogen, etc.), and further deterioration of endothelial function compared to patients with single-territory AS [5]. These factors may all play a certain role in the poorer prognosis observed in LEAD patients.

While RAS patients have a higher incidence rate of acute cardiovascular events [29], and approximately 10% of ICVD patients have concomitant RAS [8], consistent with our findings (9.7%), previous studies have not extensively explored the relationship between RAS and the risk of vascular events in ICVD patients. Our findings suggested that a higher renal AB was associated with an increased incidence of vascular events, with patients having combined RAS showing the highest incidence of vascular events. This implies that RAS also exerts an adverse effect on the prognosis of ICVD patients. One possible explanation is that reduced renal perfusion resulting from RAS can activate the Renin–Angiotensin–Aldosterone System (RAAS). An imbalance or overexpression of RAAS not only fosters the pathophysiological processes of hypertension, IS, and coronary artery disease but is also closely related to the formation and rupture of AS plaques [30]. Angiotensin II, as a product of RAAS activation, induces oxidative stress in the vascular system and plays a pivotal role in endothelial dysfunction and lipoprotein oxidation. It induces the expression of adhesion molecules and cytokines, thereby instigating the inflammatory process within the vascular wall [30]. Moreover, it might be associated with acute complications of AS by increasing plaque vulnerability and causing plaque rupture [31]. These mechanisms may contribute to the elevated incidence of vascular events observed in patients with a high renal AB, but the specific pathophysiological mechanisms remain to be further investigated.

The above findings suggested that beyond the shared pathophysiological mechanisms of AS, AS in distinct vascular beds may also increase the occurrence of vascular events through specific pathways, subsequently influencing patient prognosis. Therefore, screening for lower extremity and renal artery AS in ICVD patients may provide additional clinical insights. By calculating CPAB using CTA and vascular ultrasound, it is possible to provide a comprehensive assessment of the severity and extent of AS in multiple arterial territories non-invasively and conveniently while ensuring comparability of AS among different vascular beds and individuals. Our results indicate that CPAB offers incremental prognostic value beyond single-territory burden. Patients with higher CPAB scores tend to have worse prognoses, supporting the potential value of incorporating multiterritorial assessment into existing risk stratification models. Patients with higher CPAB scores may benefit from more stringent lipid management and comprehensive control of other vascular risk factors [3], although the specific targets remain to be determined. In the future, with advancements in non-invasive imaging technologies and artificial intelligence, this multiterritorial AB evaluation method is expected to provide precise quantitative techniques and effective risk stratification and prognostic models for developing ICVD clinical decision-support systems, as well as improving treatment monitoring.

Our study has several limitations. Firstly, it was a single-center cohort study with a majority of male patients and relatively mild neurological deficits, which may limit the generalizability of the conclusions. Secondly, the average follow-up duration was relatively short. Although the incidence of the primary endpoint was 10.7%, which may represent a clinically meaningful event rate, the limited duration and number of endpoint events remain important constraints. The impact of a high CPAB on the long-term prognosis of ICVD patients remains uncertain, necessitating further investigations with larger sample sizes and extended follow-up periods to corroborate our findings. Thirdly, although the attrition rate was only 5.2% and baseline characteristics of patients lost to follow-up did not differ significantly from those retained, loss to follow-up may still introduce bias and remains a potential limitation. Fourthly, while some clinical characteristics appeared to show borderline statistical significance in univariate Cox regression analysis, they did not remain an independent predictor in multivariate models. This suggests that the prognostic relevance of such characteristics might be limited. Moreover, the reasons for recurrent IS have not been ascertained, and the degree of risk factor control—such as blood pressure management, medication adherence, and actual medication use—remains unclear, which may also affect outcomes. Further elucidation of the mechanisms underlying IS recurrence as well as the impact of risk factor control and medication use could provide a more accurate assessment of the relationship between CPAB and vascular events. Additionally, we used CTA to calculate cervicocephalic AB and ultrasound for peripheral AB, and we applied a relatively simplified AB scoring system for each vascular territory. However, a unified examination method, more precise imaging techniques, or a more granular scoring scale may help improve the accuracy of the AB score and its predictive performance. Finally, our study only assessed the severity and extent of AS. Incorporating plaque characteristics into the evaluation for more precise classification, and exploring quantitative measures such as plaque volume—particularly of unstable components—may further enhance the predictive accuracy of the model.

## 5. Conclusions

Nearly one-third of ICVD patients had peripheral artery stenosis. The CPAB score, integrating AS burden from both cervicocephalic and peripheral territories, demonstrated improved risk stratification performance compared to single-territory or cervicocephalic-only assessments. Patients with elevated CPAB have higher rates of vascular events and may require more intensive treatment strategies. Integrating multiterritorial AS assessment into the routine evaluation of ICVD patients could enhance risk stratification and guide the implementation of more tailored and effective interventions, thereby improving the prognosis of ICVD patients.

## Figures and Tables

**Table 1:** General characteristics and the relationship with vascular risk.

Characteristics	Total (*n* = 382)	Primary Endpoint		Stroke Recurrence	
		*p*	HR (95%CI)	*p*	HR (95%CI)
Male	266 (69.6)	0.84	0.94 (0.49–1.81)	0.46	0.74 (0.34–1.63)
Age, y	63.0 (55–69)	0.081 ^2^	1.03 (1.00–1.06)	0.022 ^1^	1.05 (1.01–1.09)
Etiology					
IS	334 (87.4)	0.32	1.83 (0.56–5.92)	0.19	3.83 (0.52–28.28)
TIA	48 (12.6)	0.32	0.55 (0.17–1.78)	0.19	0.26 (0.04–1.92)
NHISS	1 (0–3)	0.44	1.04 (0.94–1.15)	0.24	1.07 (0.96–1.20)
History of HTN	259 (67.8)	0.81	1.08 (0.56–2.09)	0.41	1.44 (0.61–3.41)
History of DM	133 (34.8)	0.16	1.56 (0.85–2.89)	0.35	1.44 (0.67–3.08)
History of IS	93 (24.3)	0.38	1.35 (0.69–2.65)	0.44	1.39 (0.61–3.17)
History of CAD	56 (14.7)	0.91	1.05 (0.44–2.50)	0.90	1.07 (0.37–3.10)
Smoking	165 (43.2)	0.81	0.93 (0.50–1.72)	0.52	0.77 (0.35–1.69)
Drinking	139 (36.4)	0.99	1.00 (0.53–1.88)	0.45	0.72 (0.37–1.66)
SBP, mmHg	145.7 ± 18.9	0.66	1.00 (0.98–1.01)	0.54	0.99 (0.97–1.01)
BMI, kg/m^2^	25.6 ± 3.4	0.55	0.97 (0.89–1.07)	0.20	0.93 (0.83–1.04)
HbA1C, %	6.6 ± 1.6	0.16	1.12 (0.96–1.32)	0.34	1.10 (0.90–1.35)
CK, IU/L	99.4 ± 206.1	0.042 ^1^	1.00 (1.00–1.00)	0.011 ^1^	1.00 (1.00–1.00)
Creatinine, umol/L	63.2 ± 15.0	0.90	1.00 (0.98–1.02)	0.21	0.98 (0.96–1.01)
Homocysteine, umol/L	17.4 ± 11.7	0.77	1.00 (0.96–1.03)	0.40	0.98 (0.93–1.03)
FBG, mmol/L	6.2 ± 2.4	0.70	1.03 (0.91–1.16)	0.79	1.02 (0.88–1.19)
Total cholesterol, mmol/L	4.0 ± 1.1	0.55	0.91 (0.68–1.23)	0.52	0.89 (0.61–1.29)
HDL-C, mmol/L	1.1 ± 0.3	0.046 ^1^	0.27 (0.07–0.98)	0.21	0.37 (0.08–1.73)
LDL-C, mmol/L	2.4 ± 0.9	0.53	0.89 (0.61–1.29)	0.28	0.77 (0.47–1.24)
Apolipoprotein A1, g/L	1.2 ± 0.2	0.73	0.75 (0.15–3.70)	0.77	1.34 (0.19–9.52)
Apolipoprotein B, g/L	0.9 ± 0.2	0.56	0.67 (0.18–2.52)	0.38	0.47 (0.09–2.55)
CRP, mg/L	5.1 ± 9.6	0.43	0.98 (0.92–1.03)	0.43	0.97 (0.89–1.05)
Fibrinogen, g/L	3.3 ± 0.9	0.95	1.01 (0.72–1.42)	0.90	0.97 (0.63–1.49)
D-dimer, ug/L	1.7 ± 4.5	0.99	1.00 (0.93–1.08)	0.50	1.03 (0.95–1.11)
Neutrophils, 109/L	4.2 ± 2.1	0.13	0.85 (0.68–1.05)	0.26	0.86 (0.66–1.12)

^1^: *p* < 0.05; ^2^: *p* < 0.1; Abbreviations: NIHSS = National Institute of Health Stroke Scale; TIA = transient ischemic attack; HTN = hypertension; DM = diabetes mellitus; IS = ischemic stroke; CAD = coronary artery disease; SBP = systolic blood pressure; BMI = body mass index; HbA1C = glycosylated hemoglobin; CK = creatine kinase; FBG = fasting blood glucose; HDL-C = high-density lipoprotein cholesterol; LDL-C = low-density lipoprotein cholesterol; CRP = C reactive protein.

**Table 2:** Incidence of endpoint events in patients with atherosclerosis in different vascular beds.

	Total (*n* = 382)	Affected Vascular Bed			Number of Affected Vascular Beds			
		CAS (*n* = 264)	LEAD (*n* = 98)	RAS (*n* = 37)	0 (*n* = 104)	1 (*n* = 123)	2 (*n* = 101)	≥3 (*n* = 54)
Primary endpoint	10.7%	13.6%	18.4%	24.3%	4.8%	7.3%	9.9%	31.5%
IS recurrence	7.1%	8.3%	10.2%	13.5%	4.8%	4.9%	5.9%	18.5%

Abbreviations: CAS = cervicocephalic atherosclerosis; LEAD = peripheral artery disease; RAS = renal artery stenosis.

**Table 3:** Performance comparison of CPAB, cervicocephalic AB, and single-territory AB COX regression models.

ABs	Primary Endpoint			IS Recurrence		
	*p*	HR (95%CI)	Harrell’s C /Somers’ D	*p*	HR (95%CI)	Harrell’s C /Somers’ D
CPAB	<0.001	2.22 (1.46–3.37)	0.678/0.357	0.013	1.90 (1.14–3.17)	0.646/0.292
Cervicocephalic AB	0.002	2.05 (1.29–3.24)	0.653/0.307	0.002	1.93 (1.09–3.41)	0.634/0.268
Cervical AB	0.002	1.79 (1.24–2.89)	0.645/0.290	0.021	1.73 (1.09–2.74)	0.634/0.268
Intracranial AB	0.011	1.66 (1.12–2.46)	0.637/0.273	0.046	1.63 (1.01–2.63)	0.633/0.266
Lower extremity AB	0.010	1.60 (1.12–2.29)	0.580/0.158	0.102	1.48 (0.92–2.37)	0.570/0.140
Renal AB	0.003	2.41 (1.36–4.28)	0.661/0.321	0.047	2.15 (1.01–4.59)	0.585/0.170

Abbreviations: CPAB = cervicocephalic–peripheral atherosclerotic burden; AB = atherosclerotic burden.

## Data Availability

Data supporting the findings of this study can be obtained from the corresponding author upon reasonable request by researchers.

## Abbreviations

The following abbreviations are used in this manuscript:

AS	Atherosclerosis
ICVD	Ischemic cerebrovascular disease
PAD	Peripheral atherosclerotic diseases
LEAD	Lower extremity arterial disease
RAS	Renal artery stenosis
IS	Ischemic stroke
MI	Myocardial infarction
AB	Atherosclerotic burden
DSA	Digital subtraction angiography
CTA	Computed tomography angiography
CPAB	Cervicocephalic–peripheral atherosclerotic burden
BMI	Body mass index
RR	Relative risk
RAAS	Renin–Angiotensin–Aldosterone system
